# High Unawareness of Chronic Kidney Disease in Germany

**DOI:** 10.3390/ijerph182211752

**Published:** 2021-11-09

**Authors:** Susanne Stolpe, Bernd Kowall, Christian Scholz, Andreas Stang, Cornelia Blume

**Affiliations:** 1 Institute for Medical Informatics, Biometry and Epidemiology, University Hospital Essen, D-45147 Essen, Germany; bernd.kowall@uk-essen.de (B.K.); imibe.dir@uk-essen.de (A.S.); 2 Department for Internal Medicine, University Hospital Cologne, D-50937 Cologne, Germany; Christian.scholz@uk-koeln.de; 3 Department of Epidemiology, School of Public Health, Boston, MA 02118, USA; 4 Institute for Technical Chemistry, Leibniz University Hannover, D-30167 Hannover, Germany; blume@iftc.uni-hannover.de

**Keywords:** chronic kidney disease, CKD unawareness, gender differences

## Abstract

Chronic kidney disease (CKD) is associated with an increased risk for cardiovascular events, hospitalizations, end stage renal disease and mortality. Main risk factors for CKD are diabetes, hypertension, and older age. Although CKD prevalence is about 10%, awareness for CKD is generally low in patients and physicians, hindering early diagnosis and treatment. We analyzed baseline data of 3305 participants with CKD Stages 1–4 from German cohorts and registries collected in 2010. Prevalence of CKD unawareness and prevalence ratios (PR) (each with 95%-confidence intervals) were estimated in categories of age, sex, CKD stages, BMI, hypertension, diabetes and other relevant comorbidities. We used a log-binomial regression model to estimate the PR for CKD unawareness for females compared to males adjusting for CKD stage and CKD risk factors. CKD unawareness was high, reaching 71% (68–73%) in CKD 3a, 49% (45–54%) in CKD 3b and still 30% (24–36%) in CKD4. Prevalence of hypertension, diabetes or cardiovascular comorbidities was not associated with lower CKD unawareness. Independent of CKD stage and other risk factors unawareness was higher in female patients (PR = 1.06 (1.01; 1.10)). Even in patients with CKD related comorbidities, CKD unawareness was high. Female sex was strongly associated with CKD unawareness. Guideline oriented treatment of patients at higher risk for CKD could increase CKD awareness. Patient–physician communication about CKD might be amendable.

## 1. Introduction

**Figure 1:**
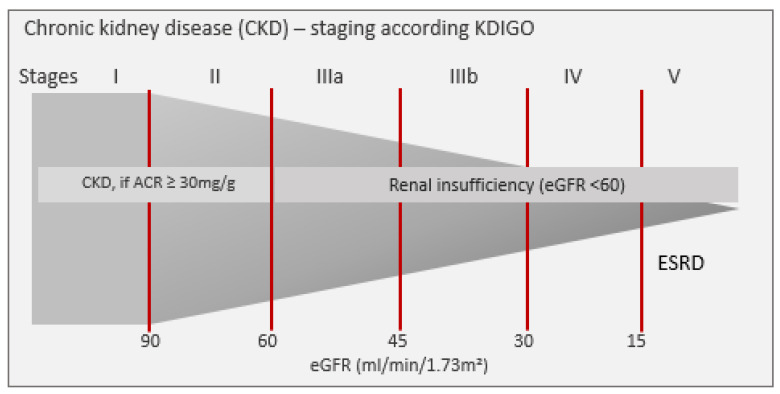
CKD staging according to decreasing renal function defined by albuminuria and estimated glomerular filtration rate (eGFR). ACR = albumin/creatinine ratio, CVD = cardiovascular disease, ESRD = end stage renal disease, KDIGO = Kidney Disease Improving Global Outcomes (=Guideline).

Chronic kidney disease (CKD) is a highly prevalent disease. Prevalence of renal insufficiency (i.e., CKD Stages 3–5) in Germany is about 10% in a population aged ≥40 years which is comparable to the prevalence of coronary heart disease, diabetes or depression [1,2,3,4]. However, CKD is widely unrecognized in its relevance for personal health consequences and its impact on societal health systems’ spending. CKD can lead to terminal renal failure (end stage renal disease, ESRD) which affects about 90,000 patients in Germany. Costs for renal replacement therapy as consequence of ESRD, such as dialysis and renal transplantation, are disproportionally high [1,5,6]. Moreover, CKD is associated with a higher risk for non-renal health outcomes such as cardiovascular diseases, hospitalizations, cognitive decline and premature mortality [5]. Kidney function is assessed by the estimated glomerular filtration rate (eGFR) using equations that incorporate serum creatinine, sex, age and race such as the CKD-Epi, Equation [7]. According to the Kidney Disease Improving Global Outcomes (KDIGO) Guideline, CKD is staged (Stages I to V) using cut points of eGFR and albuminuria (Figure 1). Patients with CKD Stage 5 (=eGFR < 15 mL/min/1.73 m^2^, (ESRD)) require renal replacement therapy.

Timely start of treatment and monitoring of CKD risk factors such as hypertension can decelerate the decrease of renal function [8]. CKD remains asymptomatic throughout a long time leading to late diagnosis in advanced stages defined by severely reduced renal function. Regarding renal insufficiency in elderly patients, discussions in the medical community whether a decline of renal function in older age should be regarded as normal physiologic aging process or be labelled as ‘disease’ are still ongoing [9].

Contrasting to diseases similarly prevalent as coronary heart disease or diabetes, for CKD, a high public and patient unawareness has been reported: unawareness was about 80% in early stages, and about 30% in later stages in populations from USA, Australia or Taiwan [10], even in patients with markers for renal dysfunction [11]. In the German Health Interview and Examination Survey for Adults (DEGS), 72% of participants—drawn from the general population—with a decreased renal function (defined as eGFR < 60 mL/min/1.73 m^2^), did not know about their condition [12]. CKD unawareness was even high in German patients hospitalized due to cardiovascular diseases [13]. Public knowledge about CKD is scarce [14]. Screening for CKD among patients and populations at higher risk was shown to be cost-effective [15], but is lacking on routine basis —resulting in late diagnosis and delayed treatment. Although eGFR should be calculated and printed automatically on patients’ laboratory reports that include measurement of serum creatinine, this seems not to lead to a routine ascertainment of kidney function and diagnosis of CKD. Physician–patient communication about CKD and its relevance for health seems to be more difficult in CKD than in other chronic diseases with negative impact on patients’ involvement and compliance to treatment [16,17].

CKD unawareness can result from either not yet being diagnosed or not being told about by a physician or not fully grasp the meaning of CKD information. 

We wanted to estimate CKD unawareness focusing on patients with CKD related risk factors, as these patients can be expected to be targeted in primary health care for monitoring renal function according to guidelines. Among these patients, we wanted to identify demographic and clinical factors that are associated with low CKD awareness. 

## 2. Materials and Methods

### 2.1. Data Source

**Figure 2:**
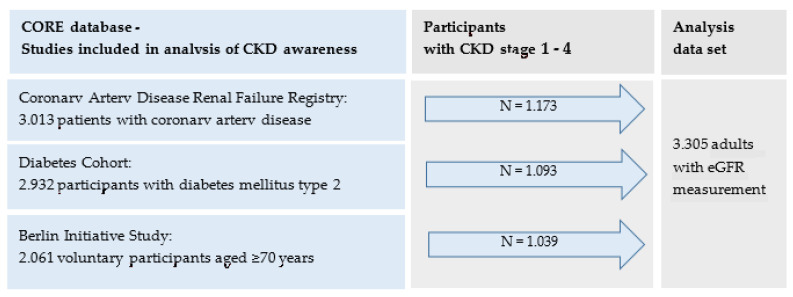
Flowchart: contributing cohorts and number of participants with CKD.

We used baseline data collected in 2010 from German CKD research cohorts and registries. In 2009, five cohorts and registries have been launched by the KfH—a German foundation for preventive medicine in regard to end stage renal kidney disease—to implore into various aspects of incidence and progression of renal insufficiency. All cohorts had the same core set of questions regarding demographic and clinical variables and laboratory measurements. These core items have been compiled to a joint database—the “CORE database” according to a standardized protocol (more information see supplementary file). For our analysis, we included participants from the diabetes cohort (DIACORE), the Coronary Artery Disease—Renal Failure Registry (CAD-Ref) and the Berlin Initiative Study (BIS). Participants in these studies either had angiographically documented coronary artery disease (CAD-Ref), self-reported diabetes mellitus type 2 (DIACORE) or were of higher age (BIS) [18,19,20], conditions that are risk factors for renal insufficiency (Figure 2). Baseline data included age, sex and clinical information such as blood pressure and BMI, questionnaire data about history of CKD relevant comorbidities, information of medication intake as well as centrally analyzed laboratory data.

For estimation of CKD unawareness, we included only those participants (N = 3305) who had a prevalent renal insufficiency or proteinuria. We categorized these participants into CKD Stages 1–4 according to the KDIGO [21] guideline definition using laboratory information on eGFR and albuminuria.

### 2.2. Variable Definition

Unknown CKD was defined according to the patients’ answer to the question ‘Have you ever been told that you have kidney disease or kidney stones?’. Laboratory data were centrally analyzed by SynLab, Germany. Serum creatinine was measured using the enzymatic method on a Roche Cobas 6000 C502, eGFR (mL/min/1.73 m^2^) was calculated using the CKD-Epi, Equation [22].

We categorized age in age groups <50, 50–59, 60–69, 70–79 and ≥80 years. 

Intake of antihypertensive medication was coded according to the patients’ answer to the respective question. Intake of antidiabetic medication was coded if participants reported taking medications with an ATC-code starting with A10, indicating anti-diabetic drugs.

An albumin/creatinine ratio (ACR) ≥ 30 mg/g indicated a micro-, an ACR ≥ 300 mg/g a macroalbuminuria. BMI was categorized in <25, 25–30, and ≥30 kg/m^2^. The KDIGO guideline 2012 recommends blood pressure lowering treatment in CKD patients with a blood pressure ≥140 mmHg systolic or ≥90 mmHg diastolic [21]. We used this threshold to define good and less good blood pressure control. We used the threshold of ≥160 mmHg systolic or ≥95 mmHg diastolic to indicate inadequate blood pressure control. Anemia was coded according to the hemoglobin value: in females with hemoglobin <12 g/dL, in males with hemoglobin <13 g/dL.

Cardiovascular disease was coded as prevalent, if a patient positively answered a question about the history of myocardial infarction, bypass surgery, stroke or heart failure.

### 2.3. Missing Values

We performed all analyses as complete-case analyses. Antihypertensive medication and smoking (N = 2), blood pressure (N = 4), BMI (N = 8), proteinuria and derived KFRE risk score (N = 74) and anemia (N = 150) had missing values.

### 2.4. Statistical Methods

We estimated the prevalence of CKD unawareness and 95% exact confidence intervals (CI) in all patients, and stratified by age, sex, CKD stage, categories of BMI and blood pressure, antihypertensive and antidiabetic medication, and comorbidities such as anemia, albuminuria, stroke, myocardial infarction or heart failure. For all selected variables, prevalence ratios (PR) for CKD awareness with 95%-CI were estimated using univariate log-binomial regression models. PRs are calculated by dividing the prevalence of unawareness in the non-reference category by the prevalence in the reference category of the respective subgroup. As we do not deal with follow-up data, it is not possible to calculate relative risk. PRs, however, can be interpreted in line with relative risks which we assume to be more intuitive than the interpretation of odds ratios. PRs less than one indicate a higher prevalence of unawareness in the reference group compared to the non-reference, PRs above one indicate a higher prevalence of unawareness in the non-reference group compared to the reference. With categorical variables, the category representing the lowest exposure was used as reference. As we want to describe associations between CKD unawareness and patients’ characteristics to depict the situation in clinical practice, adjustment for confounders is not necessary as stated by Hernan: “*if the goal of the observational analysis is purely associational, no adjustment for confounding is necessary*” [23]. As the size of presented crude PRs and their relevance may be questioned, we nonetheless present sex and age adjusted PRs for all selected CKD related risk factors. Further we estimated the PR for CKD unawareness for women compared to men in a fully adjusted model using log-binomial regression with robust error variance.

All analyses were performed in SAS 9.4.

## 3. Results

### 3.1. Characteristics of Patients

Among 3305 participants, 2267 (68%) were unaware of their CKD (Table 1). A total of 1219 (37%) patients were classified as CKD Stage 1 or 2. In 1284 (39%) patients a CKD Stage 3a and in 611 (18%) CKD Stage 3b was present. Further, 191 (5.8%) had an even more severely decreased renal function. Mean age of the participants was 73.5 (10.4 standard deviation) years, 2.5% were younger than 50 years.

Participants had a high prevalence of CKD related risk factors. Proteinuria was prevalent in 1998 (60%) patients, 2940 (89%) reported intake of antihypertensive medication. Antidiabetic medication was taken by 1495 patients (45%) and obesity was registered in 1406 (43%) patients.

Participants who were unaware of their CKD differed from those who knew about their disease in prevalence of macroalbuminuria (8.8% vs. 12.3%), anemia (20.0% vs. 32.8%) or cardiovascular comorbidities (31.1% vs. 38.3%) (Table 1). Median eGFR was higher in the unaware participants (58.4 vs. 48.4).

### 3.2. Prevalence of CKD Unawareness

Compared to men (67% (95%-CI: 65–69%)), women were more often unaware of their CKD (71% (69–74%)) (Table 2). Combining all CKD stages, unawareness decreased in older age from 78% (68–87%) in patients <50 years to 66% (63–69%) in patients aged 80 years and older.

Unawareness decreased with increasing CKD stage. A total of 82% (80–84%) of the studies’ participants with CKD Stage 1 or 2 were unaware of their CKD. In CKD Stage 3a, 71% (68–73%), and in CKD Stage 3b, 49% (45–54%) of the patients were unaware. In patients with CKD Stage 4, one out of three patients were unaware of the condition.

Even in patients with comorbidities that are risk factors for CKD or with markers of renal dysfunction, CKD unawareness was high (Table 2). About two-thirds of patients with a history of cardiovascular disease, with antihypertensive or antidiabetic medication, or with high blood pressure did not know about their CKD. Unawareness was lower in patients with macroalbuminuria (61% (56–67%)), anemia (57% (53–61%)) and in patients with at least four conditions unfavorable for CKD (60% (55–64%)). Regarding the risk for ESRD according to the KFRE equation, still 25% (18–34%) of the patients with an ESRD risk ≥15% did not know about their disease that might lead to renal replacement therapy within the next five years. 

### 3.3. Prevalence of CKD Unawareness in Regard to CKD Stages

**Figure 3:**
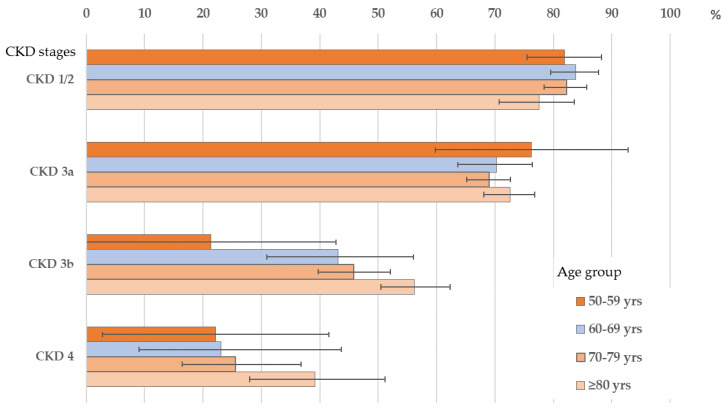
Prevalence (and 95%-CI) of unawareness in CKD Stages 1 or 2, 3a, 3b and 4 according to age group.

Stratified by CKD stage, unawareness decreased with age in CKD Stages 1–3a, but increased in later age in later CKD (Figure 3 and Supplementary Table S1).

**Figure 4:**
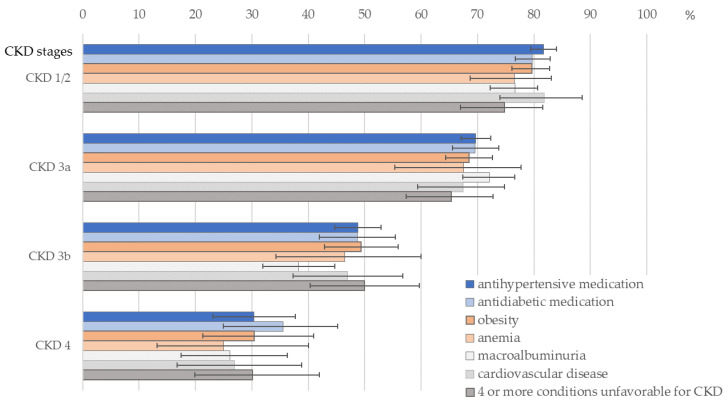
Prevalence and 95%-CI of unawareness in CKD Stages 1 or 2, 3a, 3b and 4 in subgroups of patients—conditions unfavorable for CKD: macroalbuminuria (ACR ≥ 300 mg/g), antihypertensive medication, antidiabetic medication, anemia, obesity (BMI ≥ 30 kg/m^2^), history of cardiovascular disease, current smoking, age < 70 years. More data are provided in Supplementary Table S1.

Unawareness of CKD remained high in later CKD stages (about 50% in CKD Stage 3b and 30% in CKD Stage 4) in spite of prevalent additional unfavorable health conditions such as hypertension, diabetes or overweight (Figure 4 and Supplementary Table S1).

**Figure 5:**
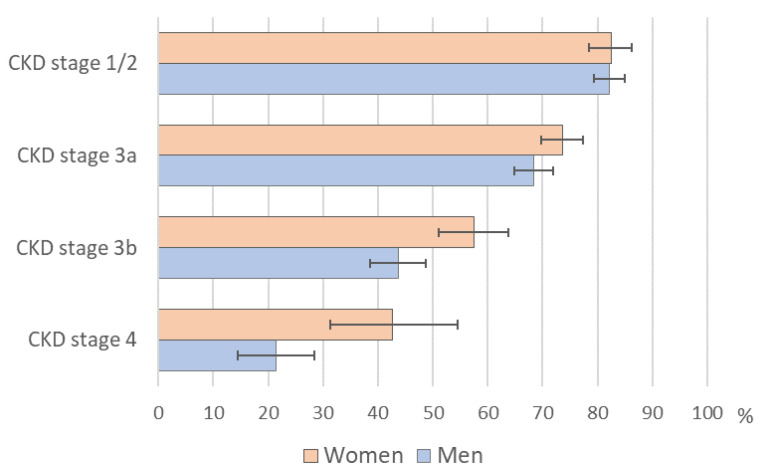
Sex difference in unawareness for chronic kidney disease (CKD) by CKD stage; prevalence of CKD unawareness with 95% confidence intervals. German CKD related cohorts (CORE database), N = 3305, 2010.

While in early CKD no sex difference in unawareness was visible, women were more often unaware of a CKD in higher CKD stages than men (Figure 5). Eventually, in CKD Stage 4, unawareness in women was twice as high as in men (43% (33–55%) vs. 22% (15–30%)). Adjusted for CKD stage and all selected CKD risk factors, female sex was independently associated with higher prevalence of CKD unawareness (PR = 1.06 (1.01; 1.10)) (see Supplementary Table S2).

### 3.4. Association between CKD Unawareness and Comorbidities and Markers of Renal Dysfunction

Anemia (PR = 0.78 (0.73–0.83)), intake of antihypertensive medication (PR = 0.85 (0.80–0.90)), macroalbuminuria (PR = 0.90 (0.82–1.00)) and history of stroke, heart failure or ischemic heart disease were univariately associated with a lower unawareness in CKD patients (Table 2). Microalbuminuria, however, was associated with a higher unawareness for CKD (PR = 1.13 (1.07–1.19)). Adjusting for all selected CKD related variables, obesity (PR = 0.94 (0.90; 0.99) and anemia (PR = 0.93 (0.87; 0.99) were independently associated with lower unawareness (Supplementary Table S2).

## 4. Discussion

In a population of patients with high prevalence of CKD risk factors, CKD unawareness was 80% in early CKD Stages 1 and 2 and still about 30% in patients with CKD Stage 4. Unawareness for CKD was seen even in the elderly or patients with hypertension or diabetes. In these patients, guidelines for treatment and drug prescribing recommend a routine monitoring of renal function. Therefore, the extent of CKD unawareness was unexpected in these subgroups and may reflect a low adherence or knowledge of guidelines. A gender gap in CKD awareness with a higher unawareness in women increased distinctly with increasing stages of CKD and was visible independently of other CKD risk factors.

### 4.1. Unawareness in Patients, Physicians and Public

Information on CKD awareness was derived from the participants’ answer to the question whether they had ever been told by their physician that they had a renal disease or kidney stones. Therefore, patients’ unawareness could either derive from a lack of understanding of the physicians’ information about their CKD or from their physicians’ unawareness of their CKD. Wagner et al. showed that hospital patients’ informational status regarding a prevalent CKD directly depended on physicians awareness [13].

In 2009, in a report on the prevalence of patients with chronic diseases in general practice, the authoring physicians did not select CKD as one of 20 relevant diseases and conditions [24], although CKD prevalence in this setting is estimated to be about 30% [25]. Guidelines on treatment of hypertension or diabetes are much more familiar in general practice than those dealing with CKD [26]. It has been shown that interventions to increase CKD knowledge and awareness in primary care physicians can lead to better CKD diagnoses and risk factor management [27]. 

In about 80% of the records of patients in general practice [28] and in hospitalized patients due to cardiovascular events [13], a prevalent CKD was not mentioned in their record, less so in patients with diabetes or obese patients. Even patients treated for CKD are often unaware. In patients from a nephrological outpatient clinic, unawareness of CKD Stage 1 or 2 was 40%, and in later stages about 12% [29]. In UK, 41% of patients with CKD Stage 3 that are documented in their GPs CKD registry were unaware of their disease [30]. 

The high CKD unawareness in patients at higher age (≥70 years) is disturbing. Age-related physiological changes in pharmacodynamics along with decreasing glomerular filtration require renal monitoring and adjustment of prescription and dosing of drugs. About 90% of the older patients in our study reported intake of antihypertensive medication. Therefore, they should regularly visit their GP. In our cohort, with increasing age, in CKD Stages 3b and 4 unawareness increased also. Physicians seem reluctant to disclose CKD related laboratory findings to their patients. As it is still discussed when to define CKD as a ‘disease’ in the elderly [9], physicians do not want to alarm their patients needlessly, when CKD is still not causing any trouble and worry about over-medicalization. However, an eGFR < 30 mL/min/1.73 m^2^ defining CKD Stage 4 should be generally regarded as pathologic. On the other hand, if physicians inform their patients about a renal dysfunction, patients may not grasp the meaning and impact it has on their health [31]. Cognitive decline which has found to be linked to CKD might negatively affect patients’ awareness [32]. However, in an analysis of primary care encounters, CKD was less often discussed than other conditions and information about CKD was given mostly about technical details as laboratory values [17,33], although better health information facilitates success in patients’ adherence to treatments [16,34]. 

### 4.2. CKD Unawareness and Additional CKD Related Risk Factors

In our cohort, in 97% of all patients, at least one condition was present which should prompt screening of renal function. However, even patients already treated or with diagnostic markers for CKD were often unaware. Albuminuria as marker for CKD seem to trigger renal screening, as unawareness was lower in these patients in our—as well as in other—cohorts [11].

Diabetes, hypertension or cardiovascular diseases were more prevalent in our cohort than in CKD patients in general [35]. Diabetic patients are at higher risk for diabetic nephropathy or other renal function disorders. Known diabetes as well as hypertension should trigger monitoring renal function. Nonetheless, unawareness was about 70% in diabetic or hypertensive patients with CKD Stage 3a and 50% in CKD Stage 3b. A German study found a similar CKD unawareness in patients with coronary heart disease [13]. US studies reported even higher CKD unawareness in patients with diabetes or hypertension [36]. In our data, still 36% of patients with diabetes and CKD Stage 4 were unaware of their CKD. A finding that is difficult to explain, as metformin, an anti-diabetic drug, is contraindicated in CKD Stage 4.

### 4.3. Unawareness and High Risk of Renal Failure

In our cohort, 21% of patients with a risk for renal failure within five years ≥15% according to the KFRE risk score were not informed about the critical state of their disease. These patients might lack necessary time for preparation for renal replacement options. In patients from NHANES with CKD Stages 3–4, unawareness was even higher among those with a KFRE risk of ≥15% (50%) [37].

### 4.4. Gender Gap in CKD Awareness

The gender gap in CKD awareness was unexpected, especially the strong increase with decreasing renal function. As unawareness for CKD in higher stages can be associated with higher probability of non-treatment or non-adherence to a treatment, women will have a higher risk for CKD-related adverse health outcomes such as cardiovascular diseases, hospitalizations, and premature mortality. In 187 participants with CKD from a German population, sex differences in CKD awareness were not visible [12]. A recent analysis of NHANES data found a higher CKD unawareness in women compared to men, but only in the Caucasian participants. Sex differences were smaller than in our cohort [38]. It is difficult to explain why women are more often unaware about a potentially critical CKD stage. Women were similarly affected by comorbidities which should require renal monitoring by a physician. Then, they would be informed about a CKD in the same manner as men. Women have been shown to be more interested in health and are more actively seeking health-related information than men [39]. Men, independent of educational attainment, are less engaged in healthy lifestyles than women [40], including exhibiting less proactive and preventive behavior [41]. Sex differences in treatment and disease outcome could be related to physicians’ bias and unconscious attitudes towards female and male patients [42].

### 4.5. Strengths and Limitations

We analyzed an exceptionally large high-quality database on CKD patients who represent a wide range of relevant comorbidities and ages. Our analyses highlight the extent of CKD unawareness in patients at higher CKD risk who might be regularly encountered in general clinical practice. 

In our analyses we defined CKD according to a single measurement of eGFR which does not fulfil the chronicity criterion for CKD and might include cases of acute kidney injury (AKI). AKI episodes occur often with acute illness, the basic examination of the participants in our dataset had not been performed due to acute health problems. Therefore, we do not expect many AKI cases to be included. Additionally, in CKD Stages 1 and 2, presence of albuminuria is necessary for diagnosis of CKD. However, CKD prevalence in patients with eGFR < 60 mL/min/1.73 m^2^ (CKD Stages 3–4) might be overestimated. As many cross-sectional epidemiologic studies are limited to a single eGFR measurement in defining CKD, the comparability of our results is facilitated [37,43,44]. 

However, we do not have information on educational attainment or social status, which might be associated with patient awareness. In German CKD patients, unawareness was not associated with educational attainment [12]. As the patients in our cohort took part in a study or register, they might have been more interested in their health than the average patient. Generally, study participants seem to have higher education than the general population [45]. Therefore, the prevalence of unawareness in CKD patients in the general population might be underestimated in our analyses.

The phrasing of the index question included kidney stones. This might lead to overestimating awareness in case that patients with unknown CKD answer the index question according to their experience with kidney stones. As kidney stones are especially prevalent in younger ages (<60 years) we do not expect a relevant effect on the proportion of CKD awareness.

Last, in our analysis, few patients aged <50 years were included as the BIS cohort recruited only persons aged 70 years and older. However, in DIACORE and CAD-Ref, the proportion of younger patients was 25% and comparable to other cohorts [43]. Proportions of unaware CKD patients in age group <50 years were 82% (DIACORE) and 92% (CAD-Ref). However, as CKD prevalence in younger ages is low, it can be expected, that overall CKD unawareness would be slightly higher with a larger proportion of younger patients.

## 5. Conclusions

CKD is even prevalent as other publicly better-known chronic diseases as diabetes or coronary heart disease. However, awareness for CKD falls short. Even in a country with universal and free health coverage, guideline-recommended monitoring of renal function in patients at higher risk for CKD does not seem to reach the practice. Most alarmingly, independent of age and other risk factors, women are more often CKD unaware than men—especially in later CKD stages. Potential explanations do require further research into gender specific patient–physician communication and CKD treatment.

## Figures and Tables

**Table 1:** Characteristics of all participants and stratified by awareness for chronic kidney disease (CKD): prevalence (N, %), mean and standard deviation (SD) or median and interquartile ranges (IQR). German CKD related cohorts (CORE database), 2010.

	Not Aware of CKD (N = 2267)	Aware of CKD (N = 1038)	Total (N = 3305)
Sex (Female)	883 (39.0)	354 (34.1)	1237 (37.4)
Age (Mean (SD))	73.0 (10.6)	74.5 (9.9)	73.5 (10.4)
<50 years	65 (2.9)	18 (1.7)	83 (2.5)
50–59 years	179 (7.9)	59 (5.7)	238 (7.2)
60–69 years	472 (20.8)	176 (17.0)	648 (19.6)
70–79 years	931 (41.1)	469 (45.2)	1400 (42.4)
80+ years	620 (27.3)	316 (30.4)	936 (28.3)
eGFR (mL/min/1.73 m^2^) (median (IQR))	58.4 (49.1–80.8)	48.4 (36.9–57.8)	55.3 (45.3–74.3)
CKD1	312 (13.8)	50 (4.8)	362 (11.0)
CKD2	691 (30.5)	166 (16.0)	857 (25.9)
CKD3a	906 (40.0)	378 (36.4)	1284 (38.9)
CKD3b	301 (13.3)	310 (29.9)	611 (18.5)
CKD4	57 (2.5)	134 (12.9)	191 (5.8)
ACR (mg/g, median (IQR)) (N = 3253)	40.7 (13.3–95.5)	37.5 (12.4–112.2)	40.0 (13.0–99.8)
ACR < 30 (=no proteinuria)	798 (35.9)	435 (43.3)	1233 (38.2)
30–299	1233 (53.4)	446 (44.4)	1679 (52.0)
≥300	195 (8.8)	124 (12.3)	319 (9.9)
BMI (kg/m^2^, Mean (SD))	29.7 (5.4)	30.0 (5.5)	29.1 (5.5)
BMI < 25	432 (19.1)	177 (17.1)	609 (18.5)
25–30	883 (39.0)	399 (38.5)	1282 (38.9)
≥30	946 (41.8)	460 (44.4)	1406 (42.6)
Current smoker	258 (11.4)	77 (7.4)	335 (10.1)
Antihypertensive medication	1979 (87.3)	961 (92.8)	2940 (89.0)
BP <140/90 mmHg	1167 (51.5)	546 (52.8)	1713 (51.9)
140/90–160/95 mmHg	593 (26.2)	271 (26.2)	864 (26.2)
≥160/95 mmHg	506 (22.3)	218 (21.1)	724 (21.9)
Antidiabetic medication	1029 (45.4)	466 (44.9)	1495 (45.2)
Comorbidities			
Anemia ^a^ (N = 3184)	433 (20.0)	326 (32.8)	759 (24.1)
Stroke, heart failure or IHD	706 (31.1)	398 (38.3)	1104 (33.4)
Sum CKD risk factors ^b^ (4+)	248 (10.9)	175 (16.9)	423 (12.8)
Risk for ESRD (KFRE) (N = 3253))			
<2%	1990 (89.4)	673 (67.0)	2663 (82.4)
2–5%	146 (6.6)	139 (13.8)	285 (8.8)
5–15%	60 (2.7)	104 (10.4)	164 (5.1)
≥15%	30 (1.4)	89 (8.9)	119 (3.7)

^a^ If female: hemoglobin < 12 g/dL, if male < 13 g/dL. ^b^ CKD risk factors comprise: macroalbuminuria (ACR ≥ 300 mg/g), hypertension, antidiabetic medication, history of stroke, heart failure or ischemic heart disease, anemia, obesity (BMI ≥ 30 kg/m^2^), current smoking and age ≥ 70. ACR = albumin/creatinine ratio, BMI = body mass index, BP = blood pressure, CKD = chronic kidney disease, ESRD = end stage renal disease, IHD = ischemic heart disease, IQR = interquartile range, KFRE = Kidney failure risk equation.

**Table 2:** Unawareness for chronic kidney disease (CKD) Stages 1 to 4 by patients’ characteristics. Crude, and age and sex adjusted prevalence ratios (PR) and 95% confidence intervals (CI) for risk of unawareness. PR for sex adjusted for age, and PR for age adjusted for sex. German CKD cohorts (CORE database), 2010.

	N	Prevalence of CKD Unawareness	Crude PR (95%-CI)	PR (95%-CI) Adj. for Age and Sex
Female	1237	71.4 (68.8; 73.9)	1.07 (1.02; 1.12)	1.07 (1.03; 1.13)
Male	2068	66.9 (64.9; 69.0)	Ref.	Ref.
Age				
<50 years	83	78.3 (67.9; 86.6)	Ref.	Ref.
50–59 years	238	75.2 (69.2; 80.6)	0.96 (0.84; 1.10)	0.96 (0.84; 1.09)
60–69 years	648	72.8 (69.2; 76.2)	0.93 (0.82; 1.05)	0.95 (0.84; 1.07)
70–79 years	1400	66.5 (64.0; 69.0)	0.85 (0.75, 0.96)	0.85 (0.76; 0.96)
80+ years	936	66.2 (63.1; 69.3)	0.85 (0.75; 0.96)	0.84 (0.75; 0.95)
CKD stages				
CKD 1	362	86.2 (82.2; 89.6)	Ref.	Ref.
CKD 2	857	80.6 (77.8; 83.2)	0.94 (0.89; 0.99)	0.93 (0.88; 0.98)
CKD 3a	1284	70.6 (68.0; 73.0)	0.82 (0.78; 0.86)	0.81 (0.76; 0.86)
CKD 3b	611	49.3 (45.2; 53.5)	0.57 (0.52; 0.63)	0.57 (0.52; 0.62)
CKD 4	191	29.8 (23.5; 36.3)	0.35 (0.28; 0.43)	0.34 (0.28; 0.43)
Proteinuria (ACR, mg/g, N = 3253)				
ACR < 30	1233	64.7 (62.0; 67.4)	Ref.	Ref.
30–299	1679	73.4 (71.3; 75.5)	1.13 (1.08; 1.19)	1.13 (1.07; 1.19)
≥300	319	61.1 (55.5; 66.5)	0.94 (0.86; 1.04)	0.94 (0.86; 1.04)
Body Weight (BMI, kg/m^2^)				
BMI < 25	609	70.9 (67.2; 74.5)	Ref.	Ref.
25–30	1282	68.9 (66.3; 71.4)	0.97 (0.91; 1.03)	0.96 (0.90; 1.03)
≥30	1406	67.3 (64.8; 69.7)	0.95 (0.89; 1.01)	0.91 (0.86; 0.97)
Current smoking				
No/Ex-smoker	2968	67.7 (66.0; 69.4)	Ref.	Ref.
Current smoker	335	77.0 (72.1; 81.4)	1.14 (1.70; 1.21)	1.10 (1.03; 1.18)
Hypertension/Blood pressure (mmHg)				
No antihypertensive medication	363	79.3 (74.8; 83.4)	Ref.	Ref.
Antihypertensive medication	2940	67.3 (65.6; 69.1)	0.85 (0.80; 0.90)	0.88 (0.83; 0.93)
BP < 140/90 mmHg	1713	68.1 (65.9; 70.3)	Ref.	Ref.
140/90–160/95 mmHg	864	68.6 (65.4; 71.7)	1.01 (0.95; 1.07)	1.01 (0.96; 1.07)
≥160/95 mmHg	724	69.9 (66.4; 73.2)	1.03 (0.97; 1.09)	1.03 (0.97; 1.09)
Diabetes				
No antidiabetic medication	1810	68.4 (66.2; 70.5)	Ref.	Ref.
Antidiabetic medication	1495	68.8 (66.4; 71.2)	1.01 (0.96; 1.05)	0.98 (0.93; 1.03)
Comorbidities				
Anemia ^a^ (N = 3184)	759	57.1 (53.4; 60.6)	0.79 (0.74; 0.85) *	0.80 (0.75; 0.86)
IHD or Heart Failure or Stroke	1104	64.0 (61.0; 66.8)	0.90 (0.86; 0.95) *	0.91 (0.87; 0.96)
4+ CKD risk factors ^b^	486	59.5 (55.0; 63.9)	0.83 (0.76; 0.91) *	0.84 (0.78; 0.92)
Risk for ESRD (KFRE) (N = 3253))				
<2%	2663	74.7 (73.0; 76.4)	Ref.	Ref.
2–5%	285	51.2 (45.3; 57.2)	0.69 (0.61; 0.77)	0.69 (0.62; 0.78)
5–15%	164	36.6 (29.2; 44.5)	0.49 (0.40; 0.60)	0.50 (0.41; 0.61)
≥15%	119	25.2 (17.7; 34.0)	0.34 (0.25; 0.46)	0.34 (0.25; 0.46)

ACR: albumin/creatinine-ratio (mg/g), BMI: body mass index (kg/m^2^), BP: blood pressure, ESRD: end stage renal disease, IHD: ischemic heart disease, KFRE: kidney failure risk equation; * reference = patients without comorbidity; ^a^: hemoglobin <12 g/dL (female), <13 g/dL (male); ^b^: CKD risk factors comprise: macroalbuminuria (ACR ≥ 300 mg/g), antihypertensive medication, antidiabetic medication, anemia, obesity (BMI ≥ 30 kg/m^2^), history of stroke or ischemic heart disease or heart failure, current smoking, age < 70 years. Interpretation of prevalence ratios using the subgroup by gender: PR = 1.07: CKD unawareness in female patients is by 7% higher than in male patients.

## Data Availability

Until now, the analyzed data have not been made publicly archived.

## Supplementary Materials

The following are available online at https://www.mdpi.com/article/10.3390/ijerph182211752/s1, S0: Characteristics of the German CORE database. Table S1: Prevalence of unawareness for chronic kidney disease (CKD) (95% confidence interval) by demographic and clinical parameters, stratified by CKD stage (N = 3305). German CKD related cohorts (CORE database, 2010), Table S2: Relative risk (and 95% confidence interval) for CKD unawareness for female compared to male patients–complete log-binomial model. Baseline data from German CKD related cohorts (CORE database), N = 3305).
