# Supplementary material for: High Unawareness of Chronic Kidney Disease in Germany
Source: Int J Environ Res Public Health. 2021 Nov 9;18(22):11752. doi: 10.3390/ijerph182211752 (PMC8623832; doi:10.3390/ijerph182211752)
Supplement: Supplementary file 1 [file ijerph-18-11752-s001.zip › ijerph-1380026-supplementary.pdf]

Content of the supplementary file:

S0. Characteristics of the German CORE database

Table S1

Prevalence of unawareness for chronic kidney disease (CKD) (95%-confidence interval) by demographic and clinical parameters, stratified by CKD stage (N=3,305). German CKD related cohorts (CORE database), 2010.

Table S2

Relative Risk (and 95%-confidence interval) for CKD unawareness for female compared to male patients – complete log-binomial model. Baseline data from German CKD related cohorts (CORE database), N=3,305.

S0. Characteristics of the German CORE database

The CORE database was launched in 2009 from the KfH-Foundation for preventive medicine of the board of trustees for dialysis and renal transplantation (KfH-Stiftung Präventivmedizin des Kuratoriums für Dialyse und Nierentransplantation e. V.).

The foundation provided scientific funding for German-wide research projects on questions on early diagnosis, disease-progression indicators and optimization of therapy of chronic kidney disease in all KDIGO-stages and patients affected by CKD of all ages. Since 2009, five cohort studies with CKD patients were financed. Four CKD studies and one registry collected data on study-specific questions and measures, but provided a standardized set of core variables including life style questions, questions on family history and underlying system diseases, health care aspects, kidney and not-kidney related organ specific symptoms, diagnose-related groups, operation and procedure keys and hospitalizations. These core variables have been transferred into the CORE database according to a standardized protocol. All laboratory data was analyzed in one central laboratory.

Follow-up time for these studies was up to 10 years with a mean of 6-7 years.

The five studies/registries within the CORE database are:

- DIACORE (DIAbetes COhoRtE): analyses of life-style and genes in order to identify parameters that trigger the emergence of adverse complications in some patients but not in the others. Included in DIACORE were patients with diabetes mellitus Type 2.

- CAD-REF (Coronary-Artery-Disease- Renal Failure): a German-wide registry including patients with a coronary artery disease. Patients with and without a CKD were included.
  The registry evaluated diagnostic markers and genetic factors using an extensive biobank for analyses of coronary diseases and (incidence of) renal insufficiency.

- BIS (Berlin Initiative Study): described the health status of elderly (70 years and older) with especially focusing on chronic kidney disease. Participants were members of a statutory health insurance in different medical practices in Berlin, representative for the general older population. Patient interviews included questions on life style, comorbidities and medications. The study set up focused on the development of an algorithm to determine the renal function in older aged patients (BIS2-equation).

- GCKD (German Chronic Kidney Disease Study): Included CKD patients in nephrological care to describe progress and consequences of CKD. Biomarker and genetic factors related to CKD which can be used for prediction of the pace of CKD progress and incidence of cardiovascular complications.
- 4C (Cardiovascular comorbidity in Children with Chronic kidney disease): analyzed severity, progression and factors leading to incident cardiovascular diseases in children with CKD. Children with CKD 3b-5 from 12 European countries aged 6-18 were included.

In our analysis on CKD unawareness, we used the variables/questions that are the same for all studies relating to clinical and demographic information as well as to laboratory measurements.

We did not include GCKD participants. These patients had already been treated for CKD before inclusion into the study and therefore know about their CKD. 4C was limited to children with CKD, therefore we did not include these participants.

Table S1. Prevalence of unawareness for chronic kidney disease (CKD) (95%-confidence interval) by demographic and clinical parameters, stratified by CKD stage (N=3,305). German CKD related cohorts (CORE database), 2010.

	CKD stage 1/2	CKD stage 3a	CKD stage 3b	CKD stage 4
Age (yrs)				
<50	87.5 (77.6; 94.1)	50.0 (0.07; 93.2)	-	-
50-59	81.9 (75.5; 87.3)	76.3 (59.8; 88.6)	21.4 (0.05; 50.8)	22.2 (2.8; 60.0)
60-69	83.8 (79.5; 87.7)	70.3 (63.6; 76.4)	43.1 (30.9; 56.0)	23.1 (9.0; 43.7)
70-79	82.3 (78.4; 85.7)	69.0 (65.2; 72.7)	45.8 (39.7; 52.1)	25.6 (16.4; 36.8)
80+	77.6 (70.7; 83.6)	72.6 (68.1; 76.8)	56.2 (50.5; 62.3)	39.2 (28.0; 51.2)
BMI (kg/m, N=6,518)				
<25	86.9 (81.7; 91.0)	70.5 (64.3; 76.1)	54.9 (45.2; 64.3)	19.4 (7.5; 37.5)
25-<30	83.3 (79.4; 86.8)	72.4 (68.4; 76.1)	46.9 (40.6; 53.2)	34.3 (23.2; 46.9)
≥30	79.6 (76.1; 82.8)	68.5 (64.3; 72.6)	49.4 (42.9; 55.9)	30.4 (21.3;40.9)
Current smoker				
No	81.7 (79.2; 84.0)	70.0 (67.4; 72.6)	49.2 (45.1; 53.4)	31.3 (24.5; 38.7)
Yes	85.2 (79.5; 89.8)	78.8 (68.6; 86.9)	50.0 (32.4; 67.6)	14.3 (1.8; 42.8)
Antihypertensive medication				
No	85.4 (79.6; 90.1)	79.1 (71.2; 85.6)	59.3 (38.8; 77.6)	20.0 (2.5; 55.6)
Yes	81.7 (79.2; 84.0)	69.7 (66.9; 72.3)	48.8 (44.7; 52.9)	30.4 (23.8; 37.7)
Blood pressure (mmHg)				
<140/90	83.8 (80.4; 86.7)	71.0 (67.4; 74.3)	49.3 (43.8; 54.7)	31.1 (22.9; 40.2)
140/90-160/95	80.6 (76.2; 85.0)	72.2 (67.2; 77.0)	47.7 (39.7; 55.9)	28.6 (16.6; 43.3)
>160/95	81.4 (76.8; 85.5)	67.3 (61.2; 73.0)	51.7 (42.3; 61.1)	26.1 (10.2; 48.4)
Albuminuria (ACR mg/g)				
<30 (no albuminuria)	-	71.3 (68.1; 74.4)	53.5 (48.0; 59.0)	37.7 (26.3; 50.2)
30-<300	83.1 (80.7; 85.3)	67.8 (62.6; 72.9)	47.1 (40.1; 54.2)	27.3 (17.0; 39.6)
≥300	76.6 (68.8; 83.2)	72.1 (59.9; 82.3)	38.3 (26.1; 51.8)	26.1 (14.3; 41.1)
Antidiabetic medication				
No	85.4 (82.1; 88.2)	71.2 (67.9; 74.4)	49.6 (44.5; 54.7)	24.5 (16.4; 34.2)
Yes	79.7 (76.5; 82.7)	69.6 (65.4; 73.6)	48.7 (42.0; 55.4)	35.5 (25.8; 46.1)
Anemia[a] (N=3,155)				
No	82.7 (80.3; 85.0)	71.5 (68.9; 74.8)	51.5 (46.3; 56.7)	35.5 (24.9; 47.3)
Yes	76.5 (68.4; 83.3)	67.5 (61.8; 72.7)	46.5 (39.8; 53.4)	25.0 (17.2; 34.3)
Stroke or IHD or heart failure				
No	82.5 (79.8; 84.9)	72.1 (69.0; 75.1)	50.8 (45.6; 56.1)	32.7 (23.5; 42.9)
Yes	81.8 (77.4; 85.8)	67.4 (62.7; 71.9)	47.0 (40.6; 53.4)	26.9 (18.2; 37.1)
Number of conditions unfavorable for CKD [b]				
0-3	83.3 (80.9; 85.5)	71.3 (68.5; 73.9)	49.1 (44.6; 53.6)	29.7 (21.6; 38.8)
4 and more	74.8 (67.0; 81.6)	65.4 (57.4; 72.8)	50.0 (40.3; 59.7)	30.1 (19.9;42.0)

ACR: Albumin/Creatinine Ratio, BMI: body mass index, CKD: chronic kidney disease, IHD: ischemic heart disease.

a If female: hemoglobin <12g/dl, if male <13g/dl

b CKD risk factors comprise: macroalbuminuria (ACR≥300), hypertension, antidiabetic medication, history of stroke, heart failure or ischemic heart disease, anemia, obesity, current smoking and age≥70.

Table S2. Prevalence Ratios (and 95%-confidence interval) for CKD unawareness for female compared to male patients – complete log-binomial model. Baseline data from German CKD related cohorts (CORE database, 2010), N=3,305.

	**Prevalence ratio (95% -CI)**
Female vs male	1.06 (1.01; 1.10)
CKD stage 1/2 (ref. for CKD stages)	-
CKD stage 3a	0.85 (0.79; 0.91)
CKD stage 3b	0.62 (0.56; 0.68)
CKD stage 4	0.39 (0.31; 0.49)
Age (every 10 yrs)	0.99 (0.97; 1.02)
Obesity[a] (yes vs no)	0.94 (0.90; 0.99)
Current smoking (yes vs no)	1.06 (1.00; 1.12)
Antihypertensive medication (yes vs no)	0.96 (0.90; 1.01)
Antidiabetic medication (yes vs no)	0.97 (0.93; 1.02)
Albuminuria[b] (yes vs no)	0.96 (0.90; 1.04)
Hypertension[c] (yes vs no)	0.99 (0.95; 1.03)
Anemia[d] (yes vs no)	0.93 (0.87; 0.99)
History of cardiovascular diseases[e] (yes vs no)	1.00 (0.95; 1.05)

a Body Mass Index ≥30kg/m²
b Albumin/Creatinine-Ratio ≥30mg/d
c Systolic blood pressure ≥140mmHg or diastolic blood pressure ≥ 90mmHg
d Hemoglobin <12g/dl in female, <13 g/dl in male
e Ischemic heart disease, stroke or heart failure.
